# Hematologic inflammatory indexes as a prognostic factor in endometrial cancer grading and staging 

**DOI:** 10.22088/cjim.14.3.443

**Published:** 2023

**Authors:** Mahdiss Mohamadianamiri, Majid Aklamli, Farzaneh Alemohammad

**Affiliations:** 1 Department of Obstetrics and Gynecology, Akbarabadi Clinical Research Development Center, Iran University of Medical Sciences, Tehran, Iran; 2 Department of Anesthesiology, Akbarabadi Hospital, Iran University of ‎Medical Sciences, Tehran, Iran; 3 Student Research Committee, School of Medicine, Tehran University of Medical Sciences, Tehran, Iran

**Keywords:** Endometrial cancer, Inflammation, Neoplasm grading, Prognostic factor

## Abstract

**Background:**

Endometrial cancer is one the most popular types of cancer in women in the world, also a common type of cancer among Iranian females. Neutrophils to lymphocytes (NLR) and platelet to lymphocyte (PLR) ratios are two practical and available indicators in endometrial cancer. We examined their correlation in these patients and determined that they could be used as a prognostic factor in grading and staging this cancer. This study takes a practical approach and recommends a screening strategy for asymptomatic women diagnosed with cancer in its early stages.

**Methods:**

Endometrial cancer patients were included in this cross-sectional study based on histological findings. NLR is known as the proportion of neutrophils to absolute lymphocytes, while PLR is known as the proportion of platelets to lymphocytes. The NLR and PLR were evaluated and their relationship to the grade and stage of cancer.

**Results:**

PLR and NLR values were calculated, and the mean values were 4.917±5.870 and 16.019±18.963, respectively. NLR and PLR were more significant in group 2 patients. Additionally, a strong and statistically significant relationship existed between these two methods (p<0.001). For the NL and PL methods, the optimal cutting point was 3.66 and 13.26, respectively. The NL method had a specificity of 0.906 and a sensitivity of 0.564. The PL and NL zones had values of 0.697 and 0.725, respectively. Although there is no remarkable difference among these areas, the AUC of PL power is slightly better than the NL method. It demonstrates that they are capable of increasing detection power by more than 50%.

**Conclusion:**

This study concluded that PLR and NLR were identified as independent prognostic items associated with the stage and grade of endometrial cancer.

Endometrial cancer is one the most popular types of cancer in women worldwide (1). Additionally, endometrial cancer is a common type of cancer in Iranian females. Endometrial cancer was prevalent in Iranian women at a rate of 2.8% per 100,000 people. This statistics has significantly increased over the last two decades (2). Numerous factors contribute to the development of endometrial cancer. For example, increasing obesity concurrently with a decrease in exogenous progesterone and increased compounded hormone therapy may be risk factors (3).

 Increased risk factors for endometrial cancer such as obesity and diabetes and a rise in the amount of postmenopausal women may justify a rise in the incidence of this cancer. This condition is more prevalent in postmenopausal women. Fortunately, it is frequently detected early on through symptoms such as menopausal bleeding or abnormal vaginal discharge (4).

The issue is that diagnosing these diseases in asymptomatic patients is challenging. In current years, various studies have been done to determine the prognostic parameters for endometrial cancer (5, 6). The most frequently considered adjuvant treatments for endometrial carcinoma have been radiation therapy and chemotherapy, both of which have historically been deemed ineffective (6). It is suggested that specific CBC panel parameters be used in the diagnosis and prognosis of the disease (7). Numerous studies have highlighted the systemic inflammatory response role in cancer compared to other factors; the role of the response of systemic inflammatory in cancer is well appointed. The neutrophil to lymphocyte ratio (NLR) and platelet to lymphocyte ratio (PLR) are two readily available and practical indicators. For instance, the proportion of lymphocytes to neutrophils serves as a diagnostic tool for determining the relationship between systemic inflammation and the immune system. This ratio indicates a favorable prognosis for various cancers (8-10). As such, we aimed to demonstrate the prognostic value of hematologic inflammatory indexes (NLR) in endometrial cancer grading and staging. This study takes a practical approach and recommends a screening strategy for asymptomatic women diagnosed with cancer in its early stages. 

## Methods

Patients with endometrial cancer who were receiving surgical therapy at Firoozgar Hospital Center were reviewed in this cross-sectional study. All patients included in this study were confirmed to have endometrial cancer based on histological findings. Exclusion criteria included hyperplasia and advanced stages of the endometrial disease before cancer, an incomplete CBC before surgery, and any medical condition known to affect CBC markers. The Iran University of Medical Sciences (IUMS) Ethics Committee accepted the study protocol. Patients' characteristics were gathered from different backgrounds, including their age, sex, prior medical history, and pathological findings such as cancer stage and grade (defined as the involvement of the myometer of the uterus). We used the FIGO staging system to determine the stage of endometrial cancer (11, 12).

**Stage I: **The cancer is contained within the uterus or womb and has not spread to other organs.

**Stage IA: **Cancer is detected exclusively in the endometrium or in less than half of the myometrium.

**Stage IB: **The tumor has invaded at least half of the myometrium. 

Additionally, the cancer was classified into three grades. We compared healthy and cancerous tissue. Typically, healthy tissue contains a variety of different cell types clustered together. When cancer appears to be healthy texture but has distinct cell groupings, it is referred to as a "differentiated" or "low-grade cancer." When cancerous texture seems remarkably different from healthy texture, it is referred to as a "poorly differentiated tumor" or a "high-grade tumor." The grade of cancer may assist the physician in predicting how quickly cancer will spread. Generally, the more slowly a disease is spread, the better the prognosis. The letter "G" denotes a stage of uterine cancer. G1: The cells have a high degree of differentiation. G2: The cells have a moderate degree of differentiation. G3: The cells have a low degree of differentiation. After analyzing preoperative hematologic factors such as neutrophil number, lymphocyte number, platelet number, and WBC number, NLR is known as the proportion of neutrophil number to total lymphocyte number; and PLR is defined as the ratio of platelet count to lymphocyte count. The PLR and NLR were evaluated and their correlation to the grade and stage of cancer. We categorized two groups of patients based on their cancer stage and tumor size.

Patients with grade 1 and stage 1A were classified as group 1, while those with grade 2 or 3 and stage 1B were classified as group 2. SPSS Statistics for Windows Version 24.0 (IBM Corp., Armonk, NY, USA) was applied for all statistical analyses. Statistical importance was defined as a p-value less than 0.05. The mean ± standard deviation (SD) of continuous variables was reported, while percentage and frequency values were used to report categorical data. Multivariate regression analysis was also performed to determine hematologic inflammatory indexes as an independent factor. The independent t-test was applied to compare continuous and parametric data between groups, whereas the ANOVA test analyzed categorical data. The relationship between the variables was investigated using Pearson correlation. Specificity, sensitivity, area under the curve (AUC), and optimal cut-off values were specified using receiver operating characteristic (ROC) curves.

## Results

**Figure 1:**
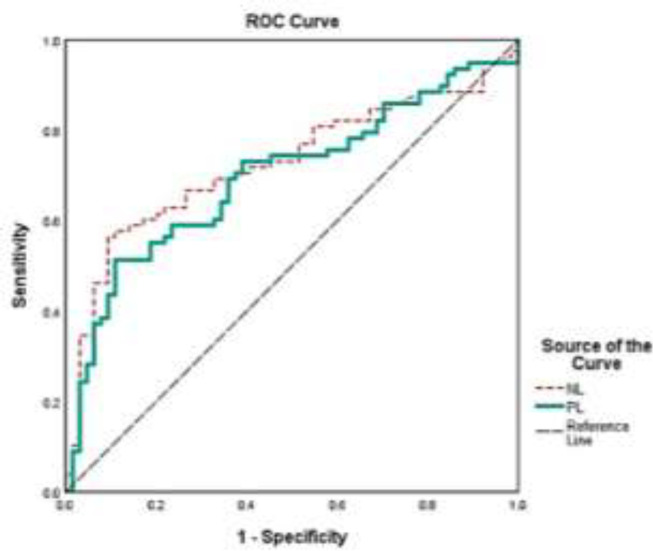
ROC curve of neutrophil to lymphocytes (NL) and platelet to lymphocytes (PL) in patients diagnosed endometrial

Endometrial cancer was diagnosed in 149 women. The mean age of patients was 60.5±12.3 years (range 13-88 years). From the total number of patients, 54.5% were classified as stage lB, while 45.5% were classified as stage lA. Additionally, 43.4% were in grade 1, 14% in grade 2, and 42.7% in grade 3. The mean±SD count of preoperative neutrophils, lymphocytes, platelets, and white blood cells were 67.5±14.1 109/L, 25.5±12.8 109/L, 253.8±87.3 109/L, and 8.25±3.2 109/L, respectively. PLR and NLR values were estimated, and the mean values were 4.917±5.870 and 16.019±18.963, respectively. The PLR and NLR were compared between group patients 1 and 2, demonstrating that these parameters were more significant in group 2. Table 1 depicts the correlation coefficients and power of NL and PL. The results indicated that the direction of the positive relationship was equal to 84. Furthermore, a strong and statistically significant relationship existed between the two methods (p<0.001). According to table 2, the combined score for sensitivity and specificity is greater than the combined score for the other points. They are chosen as the cut-point. For the NL and PL methods, the optimal cut-points were 3.66 and 13.26, respectively. The NL method showed a specificity and sensitivity of 0.564 and 0.906, respectively, and the PL method indicated a specificity and sensitivity of 0.564 and 0.906, respectively. The ROC curves for the NL and PL methods indicate that the PL and NL zones have values of 0.697 and 0.725, respectively. Moreover, the AUC values for both methods indicate that they are both above the semiconductor line. Although there is no significant difference in the area, the AUC of PL power is slightly better than the NL method. Additionally, the total NL curve is slightly steeper than the PL curve. It demonstrates that they are capable of increasing detection power by more than 50% (figure 1). 

**Table 1:** The correlation of neutrophil to lymphocytes (NL) and platelet to lymphocytes (PL) ratio in patients with endometrial cancer

**Method Area SE P-value** **Lower Bound**	**Asymptotic 95% Confidence Interval ** **Upper Bound**
NL 0.725 0.043 <0.001 0.640	0.810
PL 0.697 0.044 <0.001 0.610	0.784

NL, neutrophil to lymphocytes; PL, platelet to lymphocytes; SE, standard

**Table 2:** The cut-off point, sensitivity and specificity of NL and PL

**NL**	**PL**
**Cut-off Point** 3.66	13.26
**Sensitivity** 0.564	0.513
**specificity** 0.906	0.891
**Sum** 1.470	1.404
**Likelihood Ratio** 6	2.83

NL, neutrophil to lymphocytes; PL, platelet to lymphocytes

## Discussion

The purpose of this manuscript is to determine the prognostic value of preoperative PLR and NLR in endometrial cancer and their relationship to stage and cancer grade. Previous research suggested that inflammatory blood indexes could be used as independent prognostic items for staging and grading certain types of cancer, including colorectal cancer (13), osteosarcoma (14), head and neck cancer (15), gastric cancer (16), breast cancer (17), and lung cancer (18). Our findings indicate that PLR and NLR can be applied as independent prognostic factors to estimate the poor prognosis of endometrial cancer patients. Furthermore, we established a statistically significant relationship between these markers and cancer stage and grade.The majority of previous studies on endometrial cancer and the relationship between PLR and NLR discovered a statistically significant relationship between high NLR and cancer grading and staging but not a relationship between PLR and cancer. Pergialiotis et al. demonstrated that NLR levels were significantly elevated in endometrial cancer patients (MD 0.73, 95% CI 0.01, 1.45) (19). Additionally, Cummings et al. stated that increased PLR and NLR levels were significantly associated with benefits (P<0.001) (20). Another study by Sevgi et al. demonstrated that reduction in PLR and PDW could be used as cancer predictors. In multivariate analysis, advanced age (>53 years), low PDW (≤48.9%), and low PLR (≤133.3) were associated with a statistically significant odds ratio for diagnosing endometrioid carcinoma (21). Our study, in contrast to previous research, identifies this relationship in PLR as well as NLR. The current research findings may be beneficial in predicting the stage and grade of cancer. NLR and PLR from the patients' most recent preoperative CBC can be enhanced, and this may be a more practical and straightforward method of prognosticating the severity of cancer than other methods available, such as biopsy. Haruma et al. (22) conducted a similar cohort study in 2015 on 320 cases of endometrial cancer and identified high NLR as an independent prognostic factor, whereas our study identified PLR as an independent prognostic item for endometrial carcinoma; however, our sample size was 149 patients. Both methods have diagnostic power that is acceptable for the region. The findings of Cong et al. matched our study results (23). High NLR over 2.14 (HR = 2.71, 95% CI = 1.83-4.02, p<0.001), high PLR over 131.82 (HR = 2.75, 95% CI = 1.90-3.97, p< 0.001), and high MLR over 0.22 (HR = 1.72, 95% CI = 1.20-2.45, P = 0.003) were all significantly associated with worse overall survival, according to multivariate analyses. The combination of a high NLR + a high PLR + a high MLR (HR = 4.34, 95% CI = 2.54-7.42, p<0.001) demonstrated the best prognostic value. Kurtoglu's study (24) suggested that mean platelet volume (MPV) and platelet distribution width (PDW) are useful factors for predicting benign and malignant endometrium diseases and discriminating them. In this study, the best cut-off for differentiating benign and malignant endometrium diseases was 7.54 for MPV (odds ratio: (3.736, 95% CI = 1.865-7.486, P0.001) and less than 37.75 for PDW (odds ratio: 41.725, 95% CI = 5.426-320.868, p<0.001) were found in malignant group. There are no hematological parameters that significantly differ between endometrium cancer stages. Our study found a correlation between higher PLR and NLR values and advanced stages of cancer in one group with negative and positive lymphovascular space invasion (LVI). We determined NLR as a prognostic factor for estimating survival and that NLR and PLR have a predictive value for estimating lymph node involvement and invasion into the cervix. Furthermore, previous research has identified preoperative PLR as an independent risk factor for advanced pancreatic, colorectal, and ovarian cancers. Lin et al. demonstrated that an elevated preoperative PLR might have a predictive value in patients with colorectal cancer (25). Wang et al. concluded that preoperative PLR and NLR should be further evaluated as prognostic indicators in ampullary carcinoma resected for a cure. Moreover, it is a possible predictor of lymph node metastasis (26). Based on their NLR or PLR cut-off values, we can divide patients into two groups: those with a high NLR or PLR and those with a low NLR or PLR. This value indicated the prognosis of the disease in its advanced stages; thus, we can administer the most effective treatment. These values are deemed to be acceptable for the region. In line with our findings, Jiang et al. demonstrated that PLR levels greater than the cut-off were associated with a worse prognosis in ovarian cancer [OS: HR 1.80 (95% CI = 1.37-2.37), P = 0.000; PFS: HR 1.63 (95% CI = 1.38-1.91), P = 0.000] and cervical cancer [OS: HR 1.36 (95% CI = 1.10-1.68), P = 0.005; PFS: HR 1.40 (95% CI = 1.16-1.70), P = 0.002], however, not in cases of endometrial cancer [OS: HR 1.95 (95% CI = 0.65-5.84), P = 0.234] (4). Additionally, higher NLR and PLR values were associated with advanced disease stage, deep myometrial invasion, cervical involvement, lymphovascular space invasion (LVSI), and nodal involvement in the Temur et al.’s study. The presence of NLR was discovered to be a prognostic factor for survival (P = 0.01) (27). By examining the curves of the two methods NL and PL, it was determined that both curves are above the semiconductor line, indicating that both methods have increased detection power by more than 50%. The result indicated that the total NL curve is slightly steeper than the PL curve. NLR and PLR were discovered to be independent prognostic factors in this study. They have a statistically significant relationship with the endometrial cancer stage and grade. Furthermore, we can manage these patients more effectively and efficiently because the laboratory parameters are inexpensive and straightforward. As a result, they may be helpful as additional diagnostic and prognostic parameters in endometrial cancer
